# Impact and Interrelationships of Striatal Proteins, EPHB2, OPRM1, and PER2 on Mild Cognitive Impairment

**DOI:** 10.1007/s12035-024-04334-x

**Published:** 2024-07-13

**Authors:** Nicole Bon Campomayor, Hee Jin Kim, Hyun Jun Lee, Leandro Val Sayson, Darlene Mae D. Ortiz, Eunbi Cho, Dong Hyun Kim, Se Jin Jeon, Bung-Nyun Kim, Jae Hoon Cheong, Mikyung Kim

**Affiliations:** 1 Department of Pharmacy, Uimyung Research Institute for Neuroscience, Sahmyook University, Hwarangro 815, Nowon-gu, Seoul, 01795 Republic of Korea; 2 Department of Chemistry & Life Science, Sahmyook University, Hwarangro 815, Nowon-gu, Seoul, 01795 Republic of Korea; 3 Department of Pharmacology and Department of Advanced Translational Medicine, School of Medicine, Konkuk University, Seoul, 05029 Republic of Korea; 4 Department of Pharmacology, College of Medicine, Hallym University, Chuncheon, Gangwon 24252 Republic of Korea; 5 Department of Psychiatry and Behavioral Science, College of Medicine, Seoul National University, Daehakro 101, Jongno-gu, Seoul, 03080 Republic of Korea; 6 School of Pharmacy, Jeonbuk National University, Baekje-daero 567, Jeonju-SiJeonju-si, Jeollabuk-do 54896 Republic of Korea

**Keywords:** Mild cognitive impairment, *Per2*, EPHB2, OPRM1, Amyloid beta

## Abstract

With the global increase in life expectancy, there has been a rise in the incidence of cognitive impairments attributed to diverse etiologies. Notably, approximately 50% of individuals diagnosed with mild cognitive impairment (MCI) progress to dementia within 3 years. However, the precise mechanisms underlying MCI remain elusive. Therefore, this study aimed to elucidate potential mechanisms implicated in MCI utilizing *Per2* knockout (KO) mice, which have previously been shown to have cognitive deficits. Behavioral (Y-maze, Barnes maze) and molecular (electrophysiology, RNA sequencing, western blot, and immunofluorescence) experiments were conducted in *Per2* KO and wild-type (WT) mice. *Per2* KO mice exhibited impaired spatial working memory in the Y-maze and Barnes maze. However, there were no significant group differences in hippocampal long-term potentiation (LTP) between *Per2* KO and WT mice, whereas striatal LTP in *Per2* KO mice was lower compared to WT mice. In RNA sequencing analysis, 58 genes were downregulated and 64 genes were upregulated in the striatum of *Per2* KO mice compared to WT mice. Among the differentially expressed genes, four genes (*Chrm2*, *EphB2*, *Htr1b*, *Oprm1*) were identified. Optimal expression levels of EPHB2 and OPRM1 were found to significantly enhance cognitive performance in mice. Additionally, *Per2* KO mice exhibited reduced EPHB2-NMDAR-LTP and OPRM-mTOR signaling, along with elevated amyloid beta (Aβ) levels, when compared to WT mice. However, these alterations were reversed upon administration of morphine treatment. Striatal OPRM1-mTOR signaling, EPHB2-NMDAR-LTP signaling, and Aβ expression levels may exert a combined effect on MCI under the control of *Per2* expression.

**Supplementary Information:**

The online version contains supplementary material available at 10.1007/s12035-024-04334-x.

## Introduction

Mild cognitive impairment (MCI) is characterized by cognitive decline beyond what is expected for an individual’s age and educational background [1]. It affects approximately 19% of adults aged 65 and older, with approximately 50% of MCI patients progressing to dementia within 3 years [2]. Therefore, various pharmacological interventions targeting MCI have been developed, including cholinesterase inhibitors, dopamine agonists, and glutamate regulators [2–4]. However, the mechanism of cognitive impairment remains unclear, and currently available treatments provide only symptomatic relief without addressing the underlying condition. Numerous studies have attempted to elucidate the potential mechanisms of cognitive impairment in various aspects. Some studies have reported that amyloid beta (Aβ) accumulation ultimately leads to cognitive impairment and neurodegeneration in humans and animals [5, 6]. Additionally, various signaling pathways such as the dopaminergic system, glutamatergic system, and neuroinflammatory pathway have been focused on as potential mechanisms of MCI [2–4, 7]. Furthermore, circadian rhythm and sleep intervention play an important role in progressing MCI to Alzheimer’s disease (AD) [8]. Landry et al. conducted a comprehensive review focusing on the association between circadian dysregulation and Alzheimer’s disease (AD). The authors proposed that circadian dysregulation and disrupted sleep contribute to the progression of AD pathology by promoting inflammation and Aβ deposition. Notably, both animal and human studies have consistently demonstrated a correlation between disturbances in the circadian rhythm and cognitive impairments [9–11]. Period Circadian Regulator 2 (*PER2*), a pivotal contributor to the generation of circadian rhythms, demonstrated involvement in cognitive functions among patients with MCI; in contrast, no discernible association between cognitive performances in MCI patients and CLOCK, another circadian rhythm-related gene, was observed [12]. MCI patients with *PER2* C111G allele exhibited poorer performance across multiple cognitive assessments [13]. In an animal study using *Per2* knockout (KO) mice, heightened levels of dopamine (DA) and dopamine receptor D1 (DRD1) were observed within the hippocampus, leading to diminished cognitive performances. Furthermore, the administration of the DRD1 antagonist SCH-23390 resulted in the restoration of cognitive abilities in these mice [14]. These results indicate that *Per2* expression levels may play an important role in MCI. Although the role of the hippocampus in long-term memory consolidation and spatial working memory has been long established [14–16], there is a considerable body of research implicating that the striatum is also crucial in spatial working memory [17–19]. For example, protein synthesis in the striatum is important in spatial memory consolidation [18]. Blocking protein synthesis in the striatum induced impaired spatial working memory in the Morris water maze. Taken together, this study aimed to elucidate potential mechanisms of MCI using *Per2* KO mice.

## Materials and Methods

### Animals

This study exclusively used male *Per2*^*tm1Drw*^ (-/-) and C57BL/6 J mice (age, 8–12 weeks; weight, 22–30 g), consistent with our previous studies, to ensure comparability and consistency of results by limiting variables such as hormonal fluctuations that may affect cognitive outcomes [14, 20, 21]. Heterozygous *Per2*^*tm1Drw*^ ( ±) transgenic (TG) mice [22] were obtained from the Jackson Laboratory (Stock No.010492; JAX MICE®, ME, USA), and *Per2*^*tm1Drw*^ (-/-) mice (KO) were derived by crossing heterozygous *Per2*^*tm1Drw*^ ( +/-) TG mice. C57BL/6 J mice were obtained from RaonBio Co. (Yongin, Korea) and were used as controls based on the method in previous studies comparing results in three groups of *Per2* KO, overexpressing, and WT mice [14, 20, 23]. The mice were housed in a controlled room (12 h/12 h light/dark cycles, 7 AM–7 PM, and 22 ± 2 °C), fed a standard laboratory diet, and given water *ad libitum*. All animal treatments and maintenance were performed in accordance with the Principles of Laboratory Animal Care (NIH Publication No. 85–23, revised 1985) and the Animal Care and Use Guidelines of Sahmyook University, South Korea (SYUIACUC2022-006).

### Experiment Time

Behavioral experiments and brain tissue sample collection, except for 24-h experiments, were performed between zeitgeber time (ZT) 3–7, as previously described [23, 24]. ZT 0 indicates “lights on” (e.g., 7 AM), and ZT 12 means “lights off” (e.g., 7 PM). Y-maze and brain sample collection in WT mice for 24 h were conducted at ZT 1, 5, 9, 13, and 17.

### Materials

Morphine (MF) HCl was purchased from HANA Pharm Co. (Seoul, Korea), diluted in physiological saline (0.9% w/v NaCl), and delivered via a single intraperitoneal injection 30 min before the experiments, as previously described [20, 23]. The dosages (1 mg/kg and 5 mg/kg) of MF used in this study were obtained based on previous studies [20, 25, 26].

### Behavioral Cognitive Experiments

Y-maze and Barnes maze were used to assess cognitive flexibility including short- and long-term spatial working memory of *Per2* KO and WT mice. These assays are widely recognized for their sensitivity in detecting changes in spatial learning and memory [14].

#### Y-maze

The Y-maze consists of three identical arms (45 × 10 × 20 cm) oriented at a 120° angle to each other. Each mouse was placed on the arm end of the Y-maze and allowed to explore the environment freely for 8 min. The sequence of arm entries was recorded using Ethovison XT (RRID: SCR_000441; Noldus, the Netherlands). Actual alternation was defined as the consecutive exploration of three different arms in the sequence (e.g., ABC or BAC) during the test. The percentage of spontaneous alternations was calculated as the ratio of actual alternations to the maximum number of alternations (total number of arm entries minus two) multiplied by 100 (% alternation = [(number of alternations)/(total arm entries − 2)] × 100).

#### Barnes Maze

The Barnes maze table consists of a white circular platform (90 cm) with 20 evenly spaced holes (5 cm) around the perimeter. A goal box of black plexiglass was placed under one of the 20 holes to provide a hiding area for the animal. The visuospatial cues surrounding the maze remained in the same location throughout the test period (one habituation day and two testing days). All animals were habituated to the maze 1 day prior to the assessment. Each mouse underwent a total of eight trials at 15–20-min intervals over two consecutive days (four trials/day). The position of the goal box remained unchanged for 2 days but was placed in a different position starting from the habituation day. “Latency” is defined as the time in seconds to reach the hole with the goal box, and “error” is the number of explorations of holes visited before finding the goal box was measured to assess the spatial working memory of animals. Each exploration of an incorrect hole was considered an error, provided that the mouse lowered its nose below the plane of the table surface.

#### RNA Sequencing

RNA sequencing was conducted to investigate potential pathways of cognitive impairment in *Per2* KO and WT animals. Drug-naïve *Per2* KO and WT mice (*n* = 3/group) were randomly selected and sacrificed at ZT 3–7. The striatum in each sample was isolated using the mouse brain matrix following Allen mouse brain atlas coordinates [27]. All procedures were conducted as previously described [21]. Differentially expressed genes (DEGs) were analyzed using expression profiles. Additional analysis of DEGs was performed based on Gene Ontology (GO) (http://geneontology.org/).

### Quantitative Real-time PCR (qRT-PCR)

qRT-PCR was performed to validate five target genes selected based on GO functional analysis related to cellular components: Cholinergic receptor muscarinic 2 (*Chrm2*; forward 5`- CAT TGC GGC TTT CTA TCT GC-3`, reverse 5`-TCT GGA TCT TGT TGT GCT CCA-3`), EPH receptor B2 (*EphB2*; forward 5`-CGA CGA GAA CAT GAA CAC TA-3`, reverse 5`-CCC GTT ACA GTA GAG TTT GA-3`), 5-hydroxytryptamine receptor 1B (*Htr1b*; forward 5`-CGC CGA CGG CTA CAT TTA C -3`, reverse 5`-TAG CTT CCG GGT CCG ATA CA-3`), opioid receptor mu1 (*Oprm1*; forward 5`-CAT CAA AGC ACT GAT CAC GAT TCC-3`, reverse 5`-TAG GGC AAT GGA GCA GTT TCT GC-3`), and syntaxin 1B (*Stx1b*; forward 5`-ACT CGC AGA TGA CAA AGC AAG CC-3`, reverse 5`-CTG GGT CTG TTT TGG GAG TGA GC-3`). Drug-naïve animals (*n* = 6/group) were randomly selected and sacrificed at ZT 3–7 to obtain striatum using the mouse brain matrix. Total RNA was isolated using a Trizol reagent (Invitrogen, Carlsbad, CA, USA). A Hybrid-RTM Kit (Geneall Biotechnology, Seoul, Korea) was used for further RNA purification. The total RNA concentration was determined with a Colibri Microvolume Spectrometer (Titertek-Berthold, Pforzheim, Germany). Striatal cDNA was prepared using striatal total RNA and AccuPower® CycleScript RT PreMix (Bioneer, Seoul, Korea). All processes were performed according to the manufacturer’s instructions and as previously described [21]. The reaction was run on a StepOnePlus Real-Time PCR System (Applied Biosystems, Foster City, CA), and data were analyzed using the double delta Ct (ΔΔCt, Ct is defined as the threshold cycle) method.

### Western Blotting

The expression levels of the following target proteins in the striatum of *Per2* KO and WT mice were measured using western blotting: CHRM2 (Cat# MBS178669, MyBioSource), EPHB2 (Cat# MBS606465, MyBioSource), HTR1B (Cat# MBS9143240, MyBioSource), OPRM1 (Cat# NB100-1620, NovusBio), subunit 1 of N-methyl-D-aspartate receptor (NMDAR) 1 (Cat# SMC-410D, Stressmarq), mTOR (Cat# mAB2972, Cell Signaling), p-mTOR (Cat# mAb5536, Cell Signaling), Aβ (Cat# MBS2535169, MyBioSource), PER2 (Cat# PM083, MBLBio), and anti-β-Actin (Cat# A5441, Sigma-Aldrich). All procedures were performed as previously described [14]. The membranes were incubated in primary antibodies overnight, washed three times, and incubated at 24 °C with horseradish peroxidase-conjugated anti-rabbit (Cat# 170–6515, Bio-Rad) or anti-mouse (Cat# 170–6516, Bio-Rad) secondary antibodies for 1–2 h. A ChemiDoc Imaging System (Image Lab software version 6.0, Bio-Rad, CA, USA) was used to detect proteins.

### Immunofluorescence

We performed immunofluorescence to confirm the expression levels of two selected target proteins in mice. Brain samples were extracted from drug-naïve *Per2* KO and WT mice (*n* = 5/group) after perfusion at ZT 4–5. All procedures were performed as previously described [20]. After fixation, the sliced brain samples were incubated with primary antibodies: anti-EphB2, goat polyclonal antibody (Ab) (Invitrogen Cat# PA5-47017, RRID: AB_2609043), anti-GluN1/NR1 mouse monoclonal Ab (StressMarq Biosciences Cat# SMC-410, RRID: AB_11229703), and anti- mu (μ)-delta (δ)-opioid receptor mouse monoclonal Ab (Kerafast, Inc. Cat# EMS007). Subsequently, after washing three times, the prepared samples were incubated with donkey anti-goat Alexa Fluor 488 (Thermo Fisher Scientific Cat# A11055, RRID: AB_2534102) or goat anti-mouse Alexa Fluor Plus -555 (Thermo Fisher Scientific Cat# A32727, RRID: AB_2633276). The fluorescence levels of targets were detected by a Leica TCS-SP8 confocal microscope (Wetzlar, Germany). Corrected total cell fluorescence (CTCF) was measured using ImageJ.

### Preparation of Acute Brain Slices and Electrophysiology

In field recording, a small electrode is placed in the brain region of interest, and electrical activity is recorded from the extracellular fluid surrounding the neurons. Using a McIlwain Tissue Chopper, the dorsomedial striatum and hippocampus were prepared as 300- and 400-µm-thick coronal slices, respectively. All slices were immediately incubated in bubbled artificial cerebrospinal fluid (aCSF) (24–27 °C, 95% O2/5% CO_2_) for at least 1 h before being transferred to the submersion-type recording chamber. Electrophysiological recordings were performed in aCSF containing NaCl (124 mM), KCl (3 mM), NaHCO3 (26 mM), NaH2PO4 (1.25 mM), CaCl2 (2 mM), MgSO4 (1 mM), and D-glucose (10 mM). In the dorsomedial striatal slices, field excitatory postsynaptic potentials (fEPSPs) were recorded from the dorsomedial striatum, and four trains of high-frequency stimulation (HFS, 100 pulses at 100 Hz, 10 s interval) were introduced after a stable baseline of at least 20 min to induce long-term potentiation (LTP). In the hippocampal slices, fEPSPs were recorded from the stratum radiatum in area CA1 by stimulating an electrode placed on the Schaffer collateral-commissural pathway, and two trains of HFS (100 pulses at 100 Hz, 30 s intervals) were introduced simultaneously to induce LTP. LTP was observed 60 min after HFS. Data were collected from 12 dorsomedial and 14 hippocampal slices.

### Statistical Analysis

Data were expressed as means ± standard deviations. Y-maze, qRT-PCR, CTCF, and weight data were analyzed using two-tailed *t*-tests. Barnes maze, LTP, and western blot data were analyzed using a two-way analysis of variance (ANOVA). Linear regression or nonlinear fit was used to analyze the relationship between target proteins and cognitive function or PER2 and target proteins. Initially, we employed linear regression to investigate the correlation between the expression level of the target protein and cognitive ability in the Y-maze. However, given that the relationship between protein expression and cognitive performance exhibited a nonlinear pattern, we subsequently analyzed the data using a nonlinear fit model, specifically a bell-shaped dose–response curve (concentration–response curves) [14, 28–30]. Bonferroni tests were performed as *post-hoc* tests when there was a significant group difference after two-way ANOVA. All statistical analyses were performed using GraphPad Prism v8 (San Diego, CA, USA), and* p* < 0.05 was defined as statistical significance.

## Results

### The Cognitive Impairment Observed in *Per2* KO Mice May Be Associated with Striatal LTP Instead of Hippocampal LTP

**Fig. 1:**
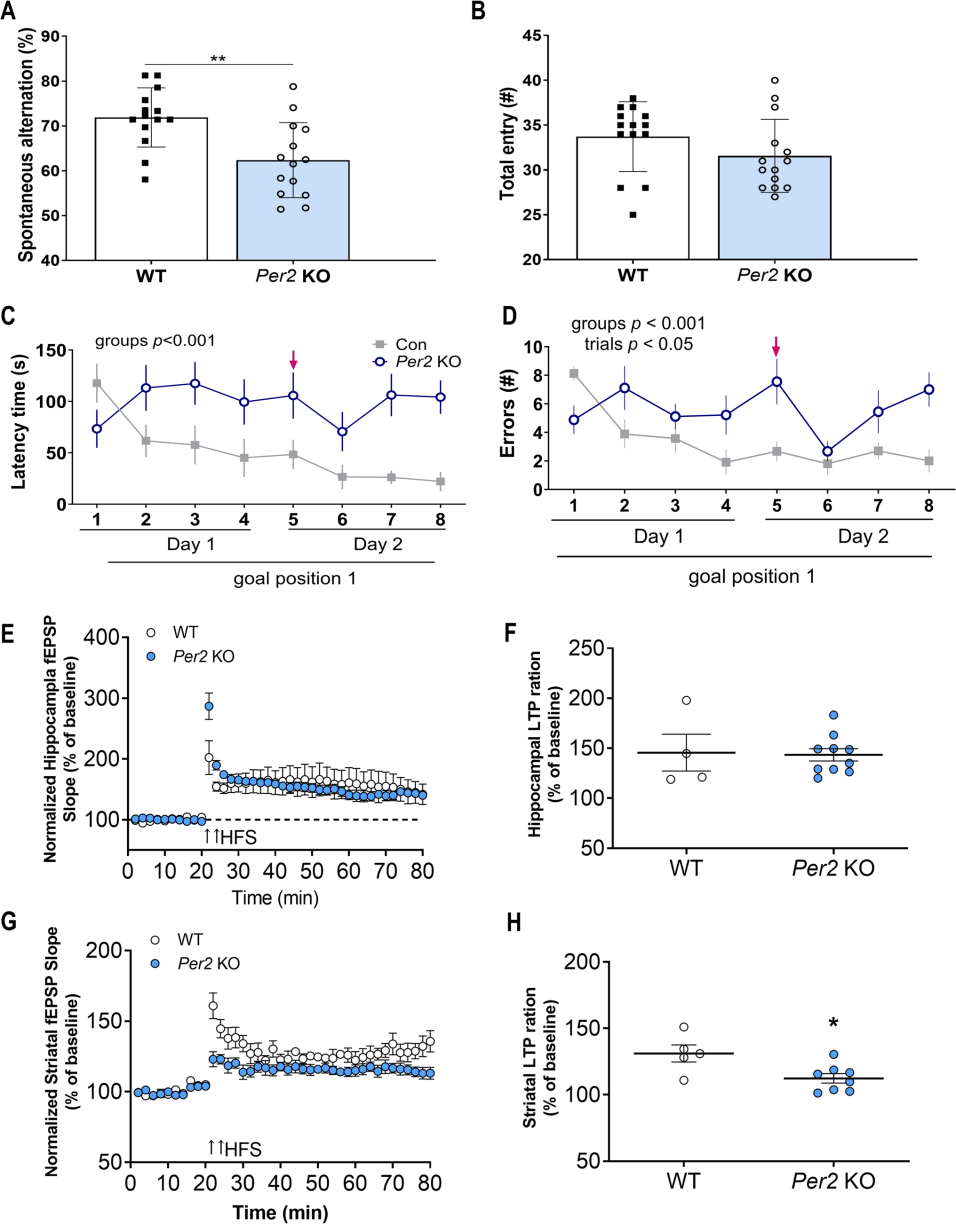
Spatial working memory of *Per2* KO and WT mice and alteration of synaptic plasticity. (**A**) Percentage of spontaneous alternation and (**B**) total entry during the Y-maze test. (**C**) Latency time and (**D**) errors in *Per2* KO and WT mice during the Barnes maze test. Red arrow indicates long-term memory on the 2nd day. (**E**) Normalized fEPSP slope before and after LTP induction in the hippocampus. To induce LTP, two trains of HFS (100 pulse, 100 Hz, 30 s interval) after 20 min baseline were applied. (**F**) Hippocampal LTP ration during last 2 min of normalized fEPSP slope. (**G**) Normalized fEPSP peak before and after LTP induction in dorsomedial striatum. To induce LTP, four trains of HFS (100 pulse, 100 Hz, 10 s intervals) after 20 min baseline were applied. (**H**) Striatal LTP ration during last 2 min of normalized fEPSP peak. *p < 0.05 and **p < 0.01, significantly different compared to the WT mice

*Per2* KO mice exhibited a significantly lower percentage of spontaneous alternation in the Y-maze compared to WT mice (*t* = 3.1, *p* < 0.05; Fig. 1A) and the Barnes maze (Fig. 1C,D). Two-way ANOVA found a significant difference in “latency” (*F*_1, 132_ = 29.16,* p* < 0.001; Fig. 1C) and “errors” (*F*_1, 128_ = 18.65,* p* < 0.001; Fig. 1D). However, there were no significant differences (*t* = 1.42, *p* = 0.17; Fig. 1B) in total entries in the Y-maze. Since LTP plays an important role in long-term and spatial memory [31], LTP was measured in the hippocampus and striatum of animals. Two-way ANOVA found no significant difference in hippocampal LTP between *Per2* KO and WT mice (*F*_1, 480_ = 0.57,* p* = 0.45; *t* = 0.15, *p* = 0.12; Fig. 1E,F). Regarding striatal LTP, there was a significant group difference between groups (*F*_1, 400_ = 111,* p* < 0.001; Fig. 1G), as LTP in *Per2* KO mice was reduced more than that in WT mice (*t* = 2.79, *p* < 0.05; Fig. 1G,H).

### Selection of Four Striatal Genes (Chrm2, EphB2, Htr1b, Oprm1) by GO Functional Analysis of *Per2* KO Showing Cognitive Impairment

**Fig. 2:**
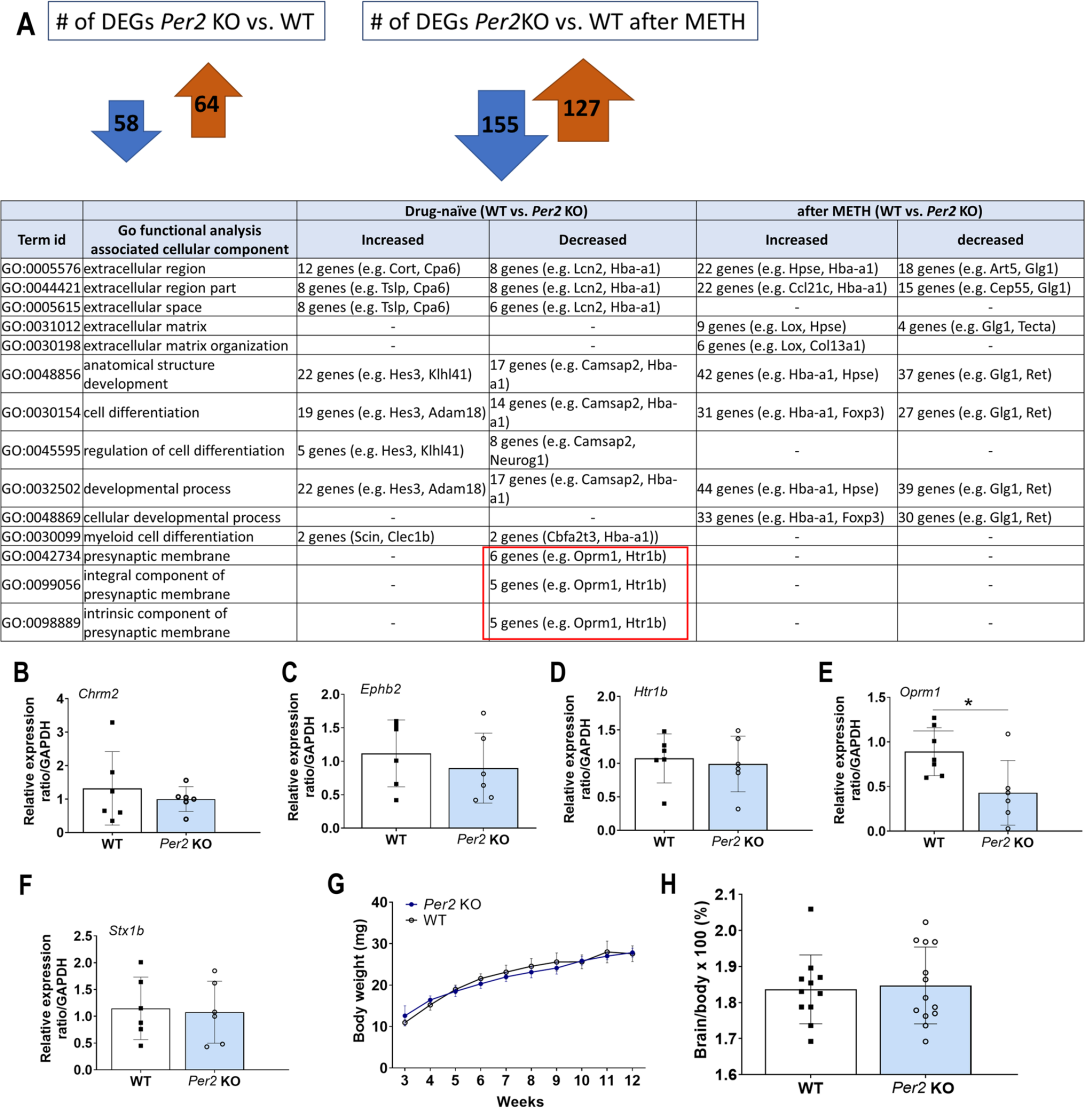
Differentially expressed genes (DEGs) in *Per2* KO and WT mice. (**A**) DEGs between *Per2* KO and WT mice before and after METH administration in GO functional analysis associated cellular components. Five genes (*Chrm2*, *Ephb2*, *Htr1b*, *Oprm1*, *Stx1b;* in red box) associated with receptors in the presynaptic membrane were downregulated only between drug-naive *Per2* KO and WT mice, with no differences after METH treatment. (**B**–**F**) The selected five genes were validated using qRT-PCR in *Per2* KO and WT mice. (**G**) Weight of *Per2* KO and WT mice. (**H**) The percentage of brain/body. *p < 0.05, significantly different by *t*-test

Based on the results of LTP experiments, RNA sequencing was conducted in the striatum. As a result, *Per2* KO mice, compared to the WT mice, exhibited 58 downregulated genes and 64 upregulated genes in the striatum (Fig. 2A). Utilizing DEGs, we explored characteristics and mechanisms related to molecular function, cellular components, and biological process in previous studies [21]. The results revealed that the top 10 molecular function GO analysis outcomes exhibited cellular characteristics such as G-protein coupled receptors (GPCR) and serotonin receptor activity. Similarly, the top 6 results of the biological process GO analysis showed cellular features related to the cellular response to monoamines or catecholamines. Based on these findings, we focused the analysis on genes impacting the cellular component GO category. Among the DEGs, only five genes (*Chrm2*, *Ephb2*, *Htr1b*, *Oprm1*, *Stx1b*) were downregulated with respect to the synaptic membrane, specifically associated with the integral (GO:0099056), intrinsic (GO:009888), and presynaptic membrane (GO:0042734) terms between *Per2* KO and WT mice, while their expression levels were recovered after METH treatment (Supplementary Table 1 and Fig. 2A). Among the five gene products, *Chrm2*, *Htr1b*, and *Oprm1* are GPCR, and *EphB2* is a kinase. GO functional analysis showed that DEGs between drug-naive *Per2* KO and WT mice were associated with GPCR, neurotransmitter receptor, and channel activities [21]. This focus was motivated by previous findings demonstrating that *Per2* KO leads to cognitive impairments through DRD1-PKA-CREB signaling changes [14], and METH treatment restores both cognitive function and gene expression levels (Fig. 2A and Supplementary Fig. 1). qRT-PCR confirmed the RNA expression levels of the five selected genes in *Per2* KO and WT mice (Fig. 2B–F). The expression levels of *Ephb2* in *Per2* KO mice tended to decrease (*t* = 1.4, *p* = 0.09; Fig. 2C), and the expression level of *Oprm1* in *Per2* KO mice was significantly decreased compared to WT mice (*t* = 2.6, *p* < 0.05; Fig. 2F). The selected genes were from the cellular components of GO functional analysis. To assess the effect of *Per2* KO on body or brain weight, the body weight of animals (once a week for 3–12 weeks) and the weight of their brains (immediately after sacrifice) were measured using a digital platform scale. As a result, there was no significant difference in body weight between the two groups (*F*_1, 322_ = 3.39,* p* = 0.07; Fig. 2G) and brain/body ratio (*F*_1, 40_ = 0.29,* p* = 0.6; Fig. 2H).

### Appropriate Expression Levels of PER2, EPHB2, and OPRM1 Were Associated with Better Cognitive Performance

**Fig. 3:**
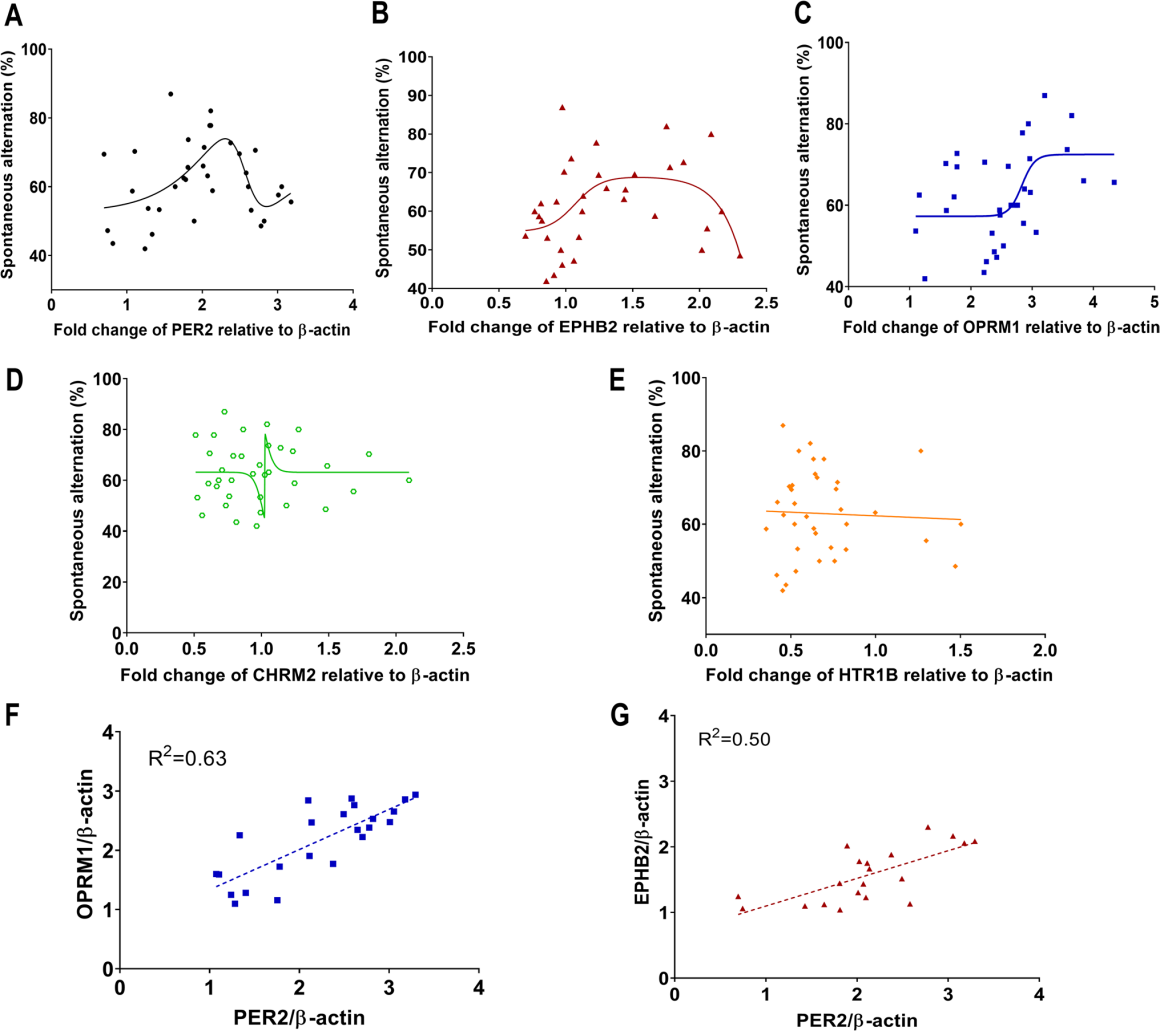
Nonlinear fit analysis of the relationship between target protein expression levels and cognitive function in the Y-maze generated using the bell-shaped dose–response curve model. (**A**) PER2 Plateau1: 66.97, Plateau2: 52.52, Peak: 10.44, LogEC50_1: 2.41, LogEC50_2: 2.564, nH1:0.95, nH2: 3.91; (**B**) EPHB2 Plateau1: ~  − 294.5, Plateau2: 54.69, Peak: ~  − 308.9, LogEC50_1: 1.07, LogEC50_2: 2.75, nH1:4.40, nH2: 2.73; (**C**) OPRM1 Plateau1: 53.51, Plateau2: 72.48, Peak: 57.29, LogEC50_1: 4.68, LogEC50_2: 2.83, nH1:80.36, nH2: -4.15; (**D**) CHRM2 Plateau1: 63.11, Plateau2: 63.15, Peak: 28.39, LogEC50_1: ~ 1.03, LogEC50_2: 1.02, nH1: ~ 697.6, nH2: 12.63; (**E**) HTR1B Plateau1: ~130.8, Plateau2: ~-22.36, Peak: ~42.2, LogEC50_1: 0.47, LogEC50_2: ~1.28, nH1:-12.64, nH2: ~-0.09;. Relationship between (**F**) EPHB2 and PER2 and (**G**) OPRM1 and PER2. Correlations were presented as fold change relative to β-actin and cognitive abilities of WT mice over 24 h seen in western blot and Y-maze test

The relationship between each target protein of the four selected genes in the striatum was assessed via western blotting and cognitive ability in the Y-maze. Initially, we conducted a linear regression analysis, which did not demonstrate a clear relationship (*R*^2^ values were low: PER2, *R*^2^ = 0.01; EPHB2, *R*^2^ = 0.04; OPRM1, *R*^2^ = 0.04; CHRM, *R*^2^ = 0.0001; Supplementary Fig. 2A–D). Therefore, we re-analyzed the results using a nonlinear fitting model. PER2 (*R*^2^ = 0.32, *n* = 35; Fig. 3A), EPHB2 (*R*^2^ = 0.23, *n* = 33; Fig. 3B), and OPRM1 (*R*^2^ = 0.29, *n* = 34; Fig. 3C) showed an inverted U-shaped relationship with cognitive ability. However, there was no inverted U-shaped trend or correlation between cognitive ability and CHRM2 (*R*^2^ = 0.15, *n* = 36; Fig. 3D) or HTR1B (*R*^2^ = not converged, *n* = 36; Fig. 3E). Based on the findings, two proteins were ultimately selected: EPHB2 and OPRM1. Finally, the correlation between PER2 and each target protein was analyzed. There was a correlation between PER2 and target proteins (EPHB2; *R*^2^ = 0.50, *p* < 0.001, *n* = 20; Fig. 3F/OPRM1; *R*^2^ = 0.63, *p* < 0.001, *n* = 23; Fig. 3G).

### Relationship Between *Per2* and the EPHB2-NMDAR-LTP Pathway

**Fig. 4:**
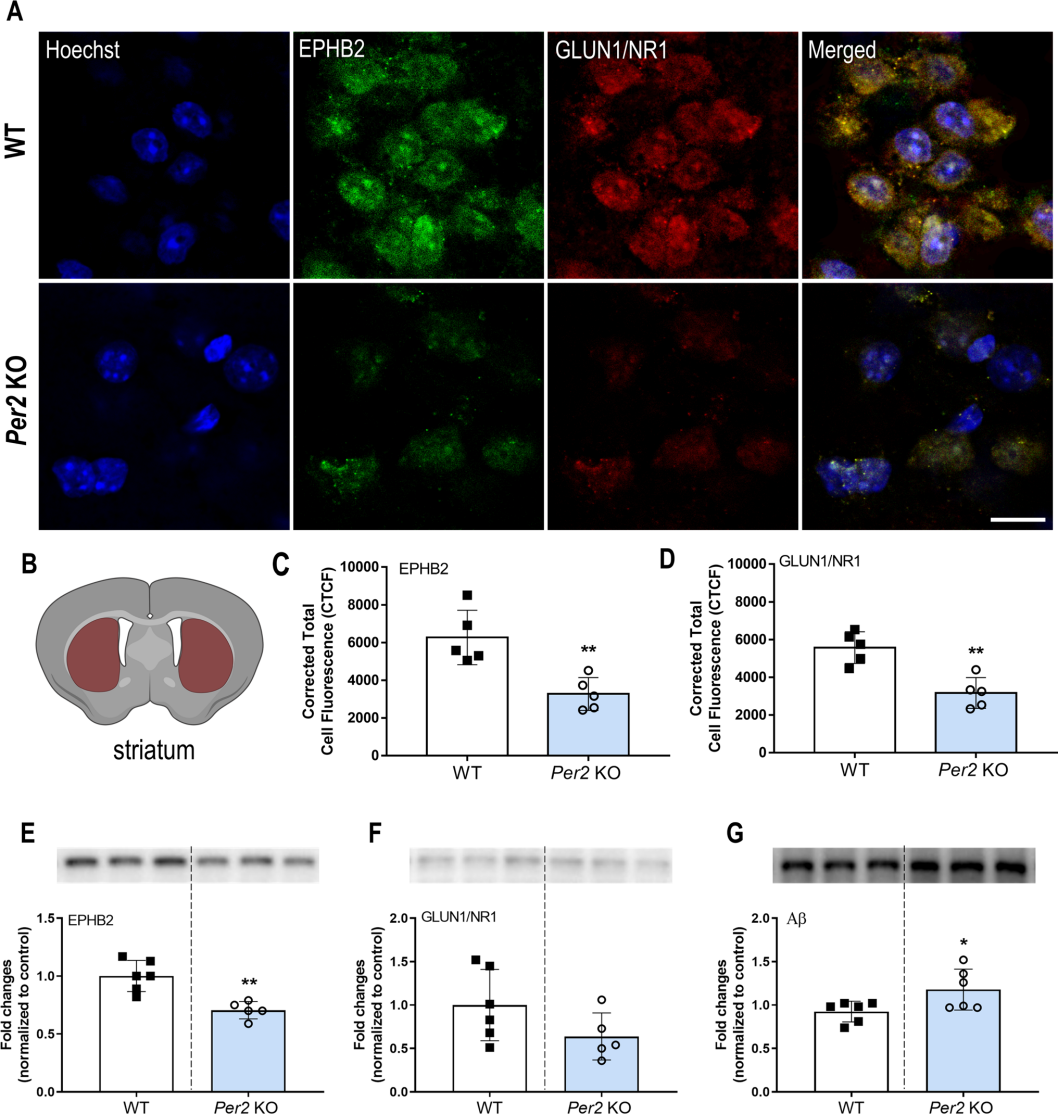
Detection and quantification of EPHB2 and GLUN1/NR1 in *Per2* KO and WT mice. (**A**) Representative immunofluorescence and corresponding corrected total cell fluorescence (CTCF) showed significantly lower fluorescence intensity of EPHB2 (**C**) and GLUN1/NR1 (**D**) antibody in the striatum (**B**) of *Per2* KO compared to WT mice. Scale bar = 10 µm. The fluorescence intensity was quantified using ImageJ (*n* = 5/group). (**E**–**G**) The expression levels of EPHB2 (**E**), GLUN1/NR1 (**F**), and Aβ (**G**) by western blotting. *p < 0.05, **p < 0.01, significantly different from the WT group

EPHB2 is involved in LTP generation through NMDAR [32, 33]. Thus, the expression levels of EPHB2 and NMDAR (GLUN1/NR1) in the striatum were assessed by western blotting and immunofluorescence in drug-naïve *Per2* KO and WT mice. CTCF showed that *Per2* KO mice had reduced EPHB2 (*t* = 3.98, *p* < 0.01; Fig. 4A,C) and GLUN1/NR1 (*t* = 4.59, *p* < 0.01; Fig. 4A,D) compared to the WT mice. Similarly, western blotting showed that *Per2* KO mice had lower EPHB2 (*t* = 4.36, *p* < 0.01; Fig. 4E,H) and GLUN1/NR1 (*t* = 1.68, *p* = 0.06; Fig. 4F,H) expression levels than did the WT mice. However, the expression level of GLUN1/NR1 was not significantly different between the two groups. To investigate the relationship between Aβ and EPHB2, the expression level of Aβ was assessed in the striatum of the mice. *Per2* KO mice exhibited a higher expression level of Aβ than did the WT mice (*t* = 2.34, *p* < 0.05; Fig. 4G,H).

### Relationship Between *Per2* and OPRM1 Activity

**Fig. 5:**
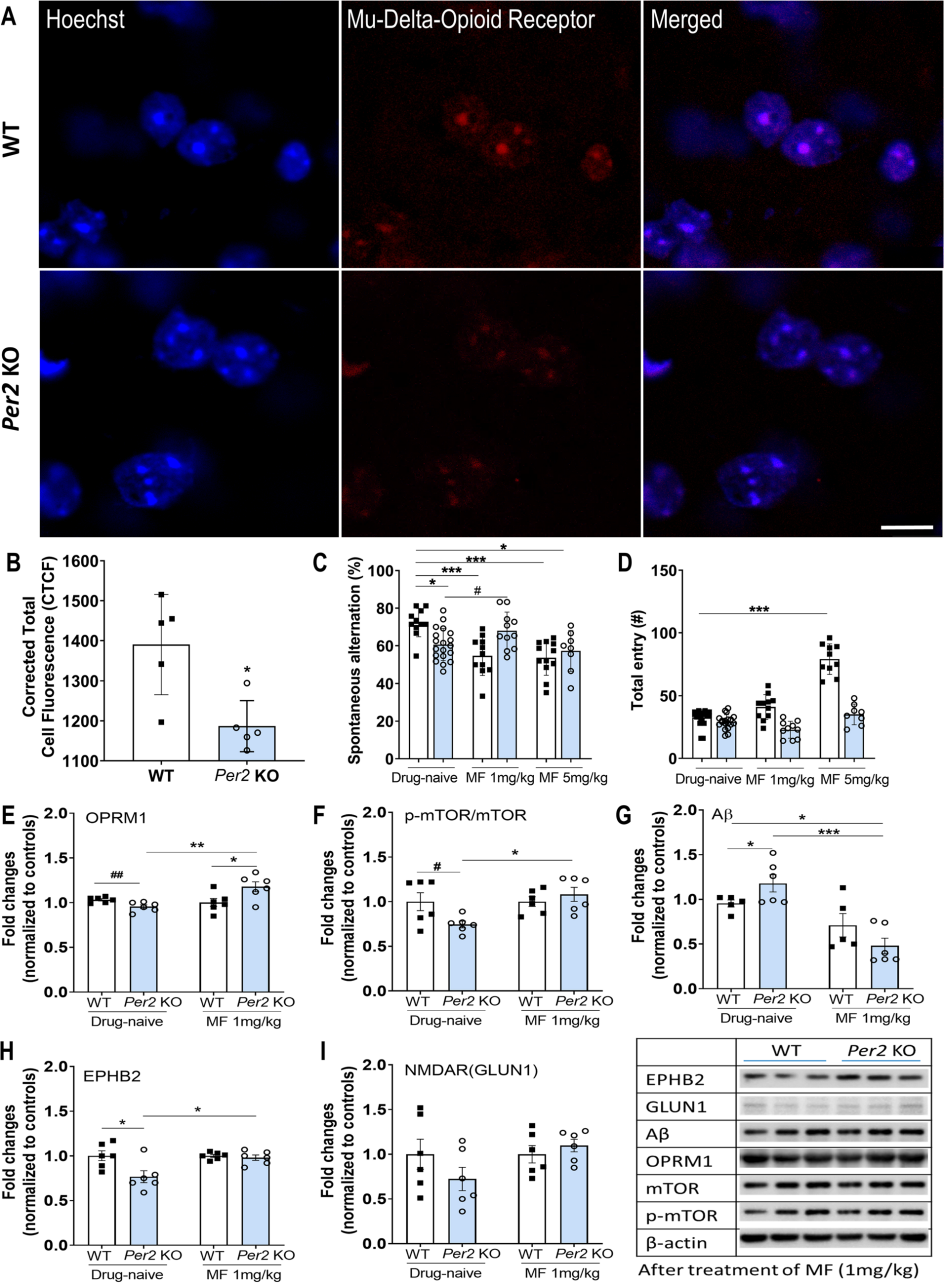
Detection and quantification of mu (μ) opioid receptor in *Per2* KO and WT mice. (**A**) Representative immunofluorescence and corresponding corrected total cell fluorescence (CTCF) showed significantly lower fluorescence intensity of μ-δ-opioid receptor (**B**) antibody in the striatum of *Per2* KO compared to WT mice. Scale bar = 10 µm. The fluorescence intensity was quantified using ImageJ (*n* = 5/group). (**C**) Percentage of spontaneous alternation and (**D**) total entry before and after morphine (1 mg/kg and 5 mg/kg) administration during the Y-maze test. (**E**–**I**) The expression levels of OPRM1, p-mTOR/mTOR, Aβ, EPHB2, and GLUN1/NR1 before and after morphine (1 mg/kg) treatment. *p < 0.5, **p < 0.01, ***p < 0.001, significantly different by two-way ANOVA. #*p* < 0.05, ##*p* < 0.01, significantly different by *t*-test

The fluorescence intensity level of μ-δ opioid receptors in the striatum of *Per2* KO mice was lower than that in the striatum of WT mice (*t* = 3.24, *p* < 0.05; Fig. 5A,B). The expression levels of OPRM1-related proteins, such as p-mTOR and Aβ, were measured by western blotting before and after morphine treatment. *Per2* KO mice showed significantly decreased expression levels of OPRM (*t* = 4.35, *p* < 0.01; Fig. 5E) and p-mTOR (*t* = 2.38, *p* < 0.05; Fig. 5F) compared to WT mice. Thus, morphine was used as a μ-opioid receptor agonist to determine whether cognitive impairment in *Per2* KO mice could be restored after morphine treatment. Cognitive ability of *Per2* KO mice was restored after morphine 1 mg/kg treatment (*t* = 2.15, *p* < 0.05; Fig. 5C), whereas morphine 5 mg/kg did not influence cognitive ability in *Per2* KO mice (*t* = 0.8, *p* = 0.43). However, WT mice exhibited cognitive impairment after treatment with morphine 1 mg/kg (*t* = 4.57, *p* < 0.001; Fig. 5C) and 5 mg/kg (*t* = 4.94, *p* < 0.001), and their number of entries increased in a dose-dependent manner (1 mg/kg; *t* = 2.85, *p* = 0.08/5 mg/kg; *t* = 15.32, *p* < 0.001; Fig. 5D). After morphine treatment, Bonferroni *post hoc* tests revealed a significant increase in the expression level of OPRM1 in the striatum of *Per2* KO mice compared to that of drug-naïve *Per2* KO mice (*t* = 4.18, *p* < 0.01; Fig. 5E) and morphine-treated WT mice (*t* = 3.31, *p* < 0.05; Fig. 5E). In addition, there was no significant difference between the two groups with respect to p-mTOR after morphine treatment (*t* = 1.14, *p* = 0.85; Fig. 5F), and the Aβ expression level in *Per2* KO mice significantly decreased after morphine treatment compared to that of drug-naïve *Per2* KO mice (*t* = 5.5, *p* < 0.001; Fig. 5G). The decreased expression levels of EPHB2 (*t* = 3.25, *p* < 0.05; Fig. 5H) and NMDAR (*t* = 3.06, *p* = 0.17; Fig. 5I) in *Per2* KO mice increased similarly to those in WT mice after morphine treatment.

## Discussion

The present study aimed to elucidate potential mechanisms of MCI using the circadian gene, *Per2*, KO mice. Our findings showed that *Per2*, *Ephb2*, and *Oprm1* collaboratively function at both RNA and protein levels to affect cognitive performance. In particular, this study validated an inverted U-shaped relationship, wherein optimal expression levels of these three target proteins were associated with enhanced cognitive ability (Figs. 3 and 5).

EPHB2 is a part of the EPHB2-NMDAR-LTP signaling pathway. It regulates NMDAR activity through tyrosine phosphorylation of NMDARs and, consequently, regulates LTP [34, 35]. *Ephb2* knockdown or KO mice had reduced NMDAR activity and impaired LTP in the hippocampus [35, 36]. In addition, increasing *Ephb2* restored NMDAR-dependent LTP of the hippocampus and cognitive abilities in AD animal models [35, 37]. Morris et al. reported that hippocampal NMDAR-dependent LTP was required for spatial working memory formation [38]. Rats treated with NMDAR blockers had impaired spatial working memory in the Morris water maze test. However, this study found that *Per2* KO mice showed similar LTP in the hippocampus as did WT mice, although *Per2* KO mice exhibited impaired spatial working memory. In contrast, *Per2* KO mice had reduced striatal LTP and expression levels of EPHB2 and NMDAR than did WT mice. The results of this study indicate that the expression level of *Per2* might influence NMDAR-dependent LTP in the striatum through the modulation of EPHB2 activity and consequently affect spatial working memory. The hippocampus is important for memory formation. However, spatial working memory involves other brain areas besides the hippocampus. For example, the prefrontal cortex and hippocampus are involved in the acquisition and encoding of spatial working memory, while the striatum is related to habit formation and consolidation of spatial working memory [18, 39–41]. In addition, spatial learning strategies and synaptic plasticity on the Barnes maze were shifted from the hippocampus to the striatum pathway by chronic alcohol exposure [42]. Therefore, even if hippocampal LTP operates normally, spatial working memory issues can still arise if the LTP of other areas, such as the striatum, is impaired. Similarly, many studies have reported that striatal LTP plays an important role in various aspects of cognition, such as spatial working memory, motor learning, attention, and decision-making. Neural plasticity in the ventral striatum is required in the spatial memory consolidation stage; in addition, NMDAR-mediated LTP in the dorsomedial striatum is involved in the acquisition and expression of instrumental conditioning tests [18, 43, 44]. For example, tissue plasminogen activator (tPA) KO mice exhibited impairment of striatal LTP [45] and hippocampal-dependent cognitive abilities [46]. This is similar to the *Per2* KO mice of the present study, showing impairment of spatial working memory and striatal LTP. A reduction in spatial working memory is a characteristic commonly observed in both aging individuals and patients with MCI [47, 48]. Therefore, *Per2* expression might influence MCI through the modulation of striatal EPHB2-NMDAR-LTP signaling.

Aβ is a peptide derived from the amyloid precursor protein (APP) and is the main component of the amyloid plaques that accumulate in the brains of AD patients [49]. Recent studies have reported the relationship between Aβ and EPHB2 signaling. Aβ binds to EPHB2 and triggers the degradation of EPHB2 in the proteasome, consequently preventing the interaction of EPHB2 with NMDARs [35]. Aβ oligomers reduced NMDARs and EPHB2 activities in hippocampal neurons [50]. In contrast, the overexpression of EPHB2 can counteract the effect of Aβ on NMDAR activity and prevent Aβ-induced neurotoxicity [50, 51]. Furthermore, this study found higher Aβ expression and lower EPHB2-NMDAR-LTP signaling activity in the striatum of *Per2* KO mice compared to those in WT mice. Taken together, the *Per2* expression level might influence cognitive activity by regulating Aβ expression and the EPHB2-NMDAR-LTP signaling pathway in the striatum.

Meanwhile, this study found that *Per2* KO mice with impaired cognitive performance had lower OPRM1 and mTOR expression and higher Aβ in the striatum than did WT mice. However, after morphine treatment (1 mg/kg), their cognitive abilities were restored, and the expression of OPRM1, mTOR, and Aβ returned to levels similar to those in WT mice. This result is consistent with that of previous studies. The activation of OPRM1 by morphine attenuated Aβ oligomers-induced neurotoxicity via the activation of mTOR, whereas OPRM1 antagonists showed opposing effects in rat cerebral cortical neurons [52]. Morphine treatment protected against Aβ toxicity in vitro and in vivo and restored impaired spatial working memory in an AD animal model [53]. In addition, several recent studies have reported that aberrant *OPRM1* methylation is associated with cognitive function in patients with MCI or AD. For example, AD patients showed elevated methylation of *OPRM1* compared to healthy people [54]. Patients with MCI in a part of China showed hypermethylation and hypomethylation of *OPRM1* [55]. Similarly, *OPRM1* polymorphism was associated with cognitive function in cancer patients [56]. In an animal study using rats, OPRM blockade in the hippocampus impaired spatial working memory in a Morris water maze [57]. The author suggested that OPRM1 and NMDAR signals, respectively, might play different roles in spatial working memory. mTOR signaling is associated with LTP and cognitive functions [58]. Aβ treatment downregulated mTOR signals in human cells [59] and suppressed mTOR activity-induced LTP impairment in the prefrontal cortex and striatum [60, 61]. Graber et al. reviewed the effects of mTOR on cognitive activity and found that mTOR signals play an important role in LTP and cognitive functions [62]. Taken together, we suggest that the OPRM1-mTOR-LTP signal might be a potential mechanism affecting cognitive ability and that the signal might be controlled by *Per2* expression. However, to the best of our knowledge, there was no clear evidence demonstrating the effect of the OPRM1-mTOR-LTP signal on cognitive ability. Besides, the effect of OPRM1 activation on cognition remains controversial. Chronic morphine exposure induced a reduction in total dendritic length and dendritic density, and high dosages of morphine impaired spatial working memory in rats [63–65]. Morphine-induced cognitive impairment varied with the dose and interval of morphine treatment [64, 65]. This might be described as an inverted U-shape. This study found that adequate expression of OPRM1 in the striatum was associated with better cognitive function, indicating irregular OPRM1 activities in the striatum might reduce cognitive activity (Fig. 5C). Interestingly, acute morphine treatment increased locomotor activity in WT mice but not *Per2* KO mice. The results in WT mice are consistent with previous studies showing that low-dose morphine treatment (1.25 mg/kg, 2.5 mg/kg, and 5 mg/kg) induced hyperactivity and high-dose morphine treatment (10 mg/kg, 20 mg/kg, and 40 mg/kg) induced hypoactivity [25, 66]. These imply that morphine may affect locomotor activity and that *Per2* may be one of the factors that inhibit the effect of morphine on locomotor activity.

**Fig. 6:**
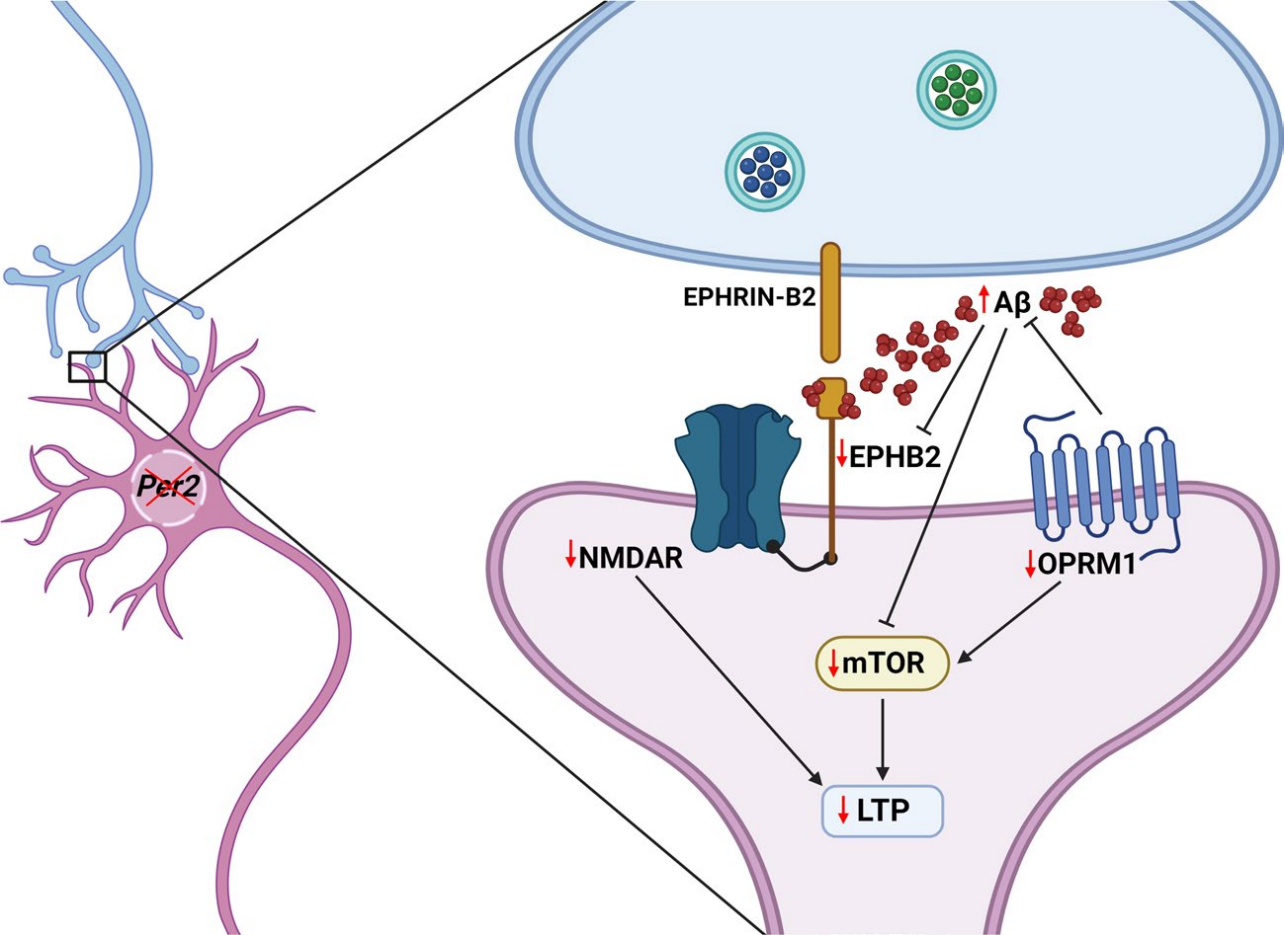
Hypothetical scheme summarizing the effect of *Per2* KO on cognitive performance. *Per2* KO mice exhibited increased Aβ levels and decreased expression levels of EPHB2, NMDAR, OPRM1, and mTOR in the striatum compared to WT mice. In addition, *Per2* KO mice showed a reduction in striatal LTP compared to WT mice. Previous studies reporting the relationship between these factors demonstrated that the expression of Aβ is inversely proportional to EPHB2, OPRM1, and mTOR; we found similar results in this study. Taken together, *Per2* expression might affect cognitive performance through the regulation of EPHB2-NMDAR-LTP signaling, OPRM1-mTOR signaling, and Aβ in the striatum. Black arrows indicate positive regulatory relationships, bars indicate inhibition, and red arrows indicate protein expression levels in *Per2* KO mice

Furthermore, the reduced expression levels of EPHB2 and NMDAR in the striatum of *Per2* KO mice were restored similarly to those of WT mice after morphine treatment in this study. To date, few studies have explicitly demonstrated the interrelationship between OPRM1 and EPHB2 in cognitive functions. A study of TG mice reported that *Ephb2* KO mice exhibited cognitive impairment in contextual learning and that *Ephb2* might regulate morphine-dependent learning and memory [67]. The authors suggested a possible molecular network between morphine-dependent changes and *Ephb2*-NMDAR-LTP signaling. Their assumption is consistent with the results of the present study. After low-dose morphine administration, the diminished expressions of EPHB2 and OPRM1 in *Per2* KO mice were restored to levels similar to, or even higher than, those observed in normal mice. Concurrently, the previously reduced cognitive abilities of the TG mice also showed signs of recovery. This study also found that the expression level of Aβ was reduced after morphine treatments. Taken together, *Per2* expression might influence spatial working memory through OPRM1-mTOR-LTP signaling, EPHB2-NMDAR-LTP signaling, and Aβ expression levels in the striatum (Fig. 6).

## Conclusion

This study explored the possibility that OPRM1-mTOR-LTP signaling, EPHB2-NMDAR-LTP signaling, and Aβ expression levels might collectively influence spatial working memory under the regulation of *Per2* expression. However, the conclusion is that interactions between EPHB2, OPRM1, and PER2 lead to cognitive impairment, which is not sufficiently supported by the observed changes in expression levels. The results of this study may provide an initial understanding of the potential interactions among these signaling pathways, and future research in this field could play a crucial role in clarifying the mechanisms of these interactions. This study provides an important foundation for investigating the effects of *Per2* expression on cognitive function but emphasizes that additional research is essential to deepen our understanding of the interactions between these signaling pathways. Further research is needed to explore these interactions in more detail and to identify potential therapeutic targets for cognitive impairments related to *Per2* dysregulation.

## Supplementary Information

Below is the link to the electronic supplementary material.

Supplementary file1 Supplementary Fig. 1 Spatial working memory of *Per2* KO & WT before and after METH administration. (**A**) Percentage of spontaneous alternation and (**B**) total entry before and after METH (0.5 mg/kg) administration during the Y-maze. (**C**) Latency time and (**D**) errors in *Per2* KO and WT mice treated with METH (0.5 mg/kg). METH administration recovered the impairments of short- and long-term memory in *Per2* KO mice (**A**, **C-D**). Red arrow indicates long-term memory on the 2nd day. *p < 0.05 and **p < 0.01, significantly different compared to the WT mice (PNG 1560 KB)

Supplementary file2 Supplementary Fig. 2 Relationship between target proteins and cognitive ability in WT mice by linear regression. Relationship between (**A**) PER2, (**B**) EPHB2, (**C**) OPRM1, and (**D**) CHRM2, and cognitive ability by linear regression. Correlations are presented as fold change relative to β-actin in western blot and cognitive abilities in the Y-maze test for WT mice. Due to the very low R² values, the linear regression analysis could not establish a significant correlation between the target proteins and cognitive ability (PER2, R^2^ =0.01; EPHB2, R^2^ =0.04; OPRM1, R^2^ =0.04; CHRM, R^2^ =0.0001) (PNG 1061 KB)

Supplementary file3 Supplementary Table 1 List of differentially expressed genes (DEGs) associated with cellular components in *Per2* KO and WT mice before and after methamphetamine treatment (XLSX 26 KB)

## Data Availability

The data sets used and/or analyzed during the current study are available from the corresponding author upon reasonable request.

## Abbreviations

Aβ: Amyloid beta
AD: Alzheimer’s disease
APP: Amyloid precursor protein
*Chrm2*: Cholinergic receptor muscarinic 2
DEGs: Differentially expressed genes
DRD1: Dopamine receptor D1
*Ephb2*: EPH receptor B2
GO: Gene ontology
GPCR: G-protein coupled receptors
*Htr1b*: 5-Hydroxytryptamine receptor 1B
KO: Knockout
LTP: Long-term potentiation
MCI: Mild cognitive impairment
MF: Morphine
*Oprm1*: Opioid receptor mu1
*Per2*: Period circadian regulator 2 gene in mice
*PER2*: Period circadian regulator 2 gene in human
PER2: Period circadian regulator 2 protein
*Stx1b*: Syntaxin 1B
WT: Wild-type
ZT: Zeitgeber time
