# Supplementary figures and images for: Impact and Interrelationships of Striatal Proteins, EPHB2, OPRM1, and PER2 on Mild Cognitive Impairment
Source: Mol Neurobiol. 2024 Jul 13;62(2):1478–92. doi: 10.1007/s12035-024-04334-x (PMC11772528; doi:10.1007/s12035-024-04334-x)

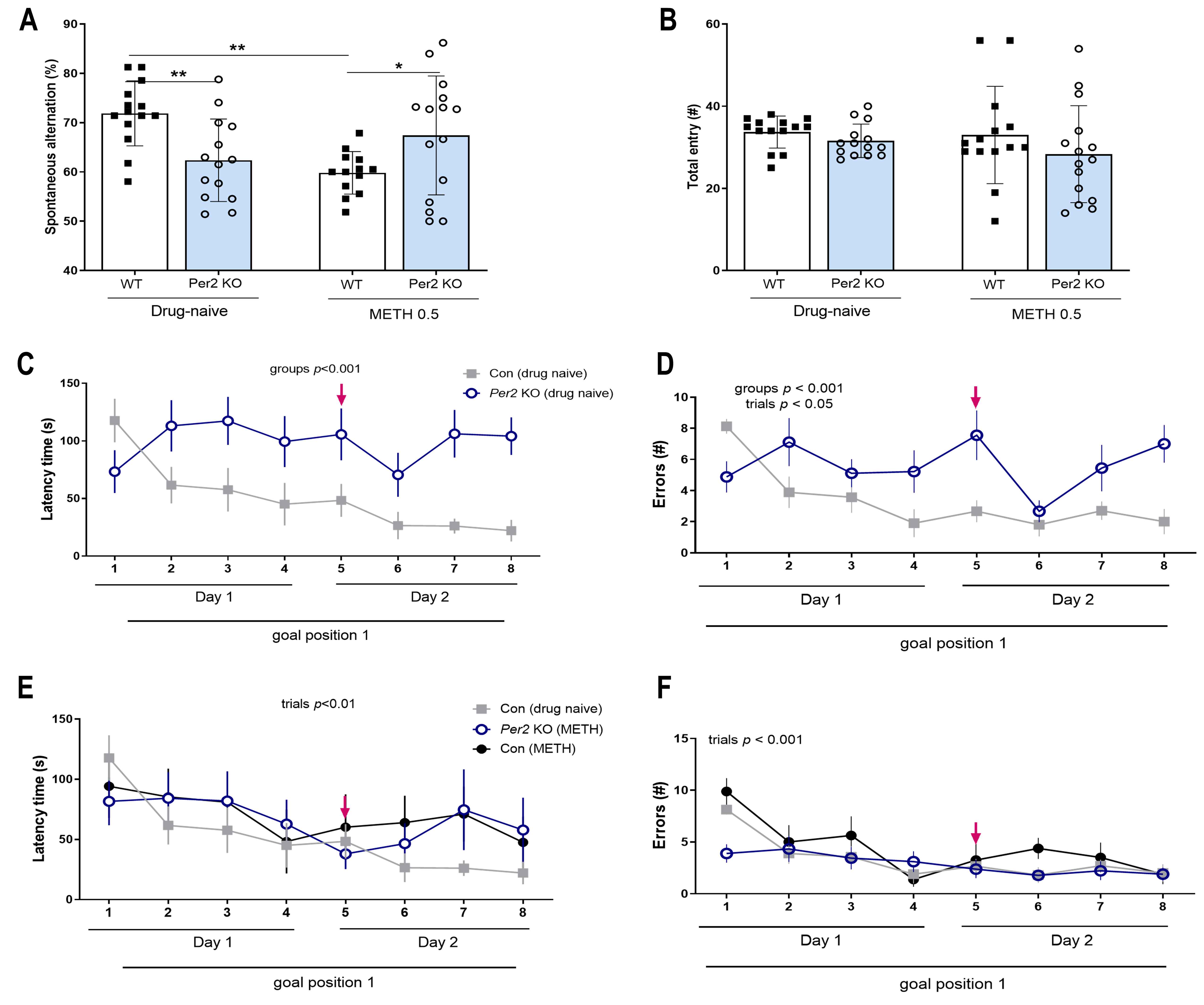

Supplement: Supplementary file 1 — Supplementary file1 Supplementary Fig. 1 Spatial working memory of Per2 KO & WT before and after METH administration. (A) Percentage of spontaneous alternation and (B) total entry before and after METH (0.5 mg/kg) administration during the Y-maze. (C) Latency time and (D) errors in Per2 KO and WT mice treated with METH (0.5 mg/kg). METH administration recovered the impairments of short- and long-term memory in Per2 KO mice (A, C-D). Red arrow indicates long-term memory on the 2nd day. *p < 0.05 and **p < 0.01, significantly different compared to the WT mice (PNG 1560 KB) [file 12035_2024_4334_MOESM1_ESM.png]

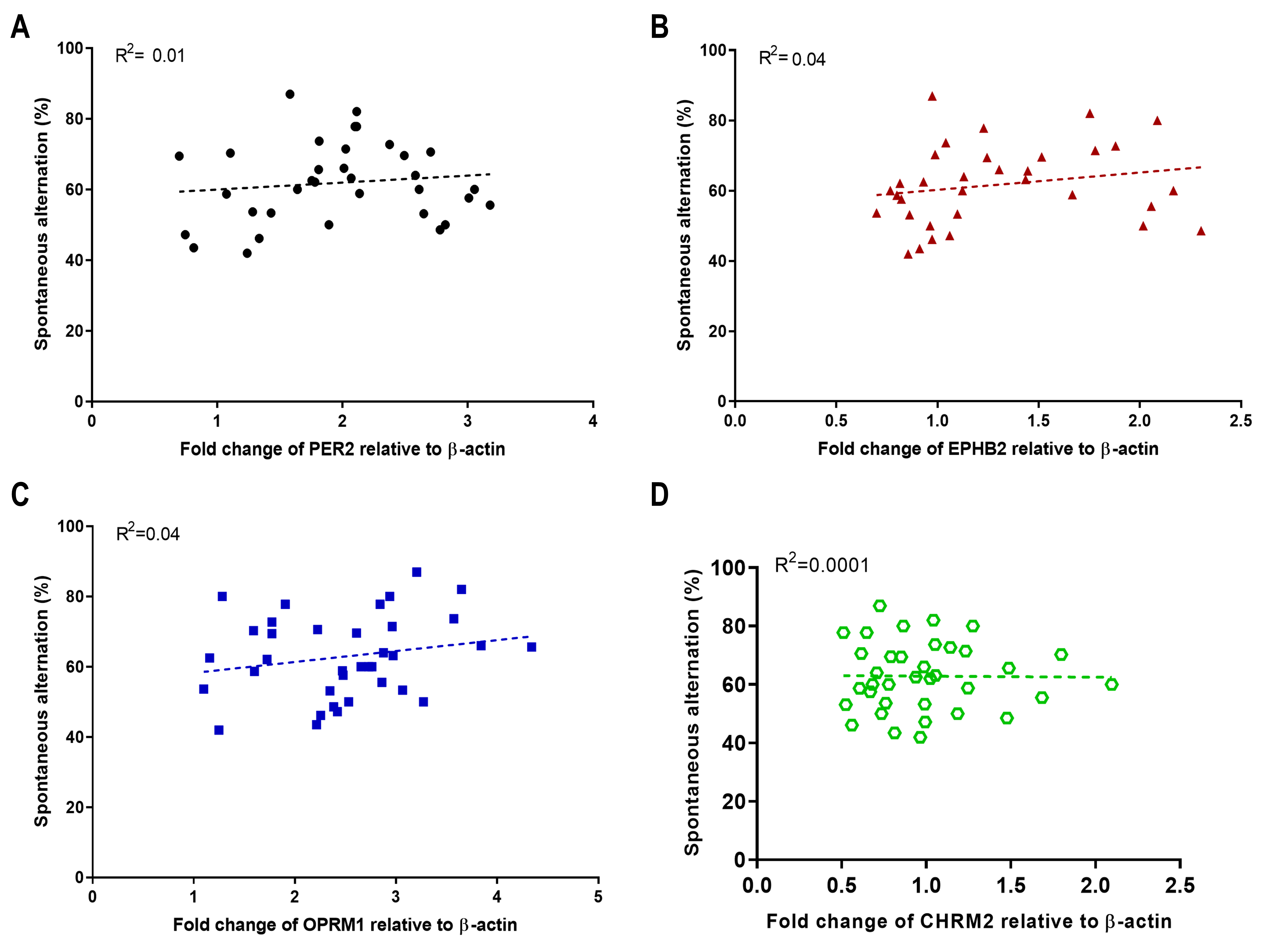

Supplement: Supplementary file 2 — Supplementary file2 Supplementary Fig. 2 Relationship between target proteins and cognitive ability in WT mice by linear regression. Relationship between (A) PER2, (B) EPHB2, (C) OPRM1, and (D) CHRM2, and cognitive ability by linear regression. Correlations are presented as fold change relative to β-actin in western blot and cognitive abilities in the Y-maze test for WT mice. Due to the very low R² values, the linear regression analysis could not establish a significant correlation between the target proteins and cognitive ability (PER2, R2 =0.01; EPHB2, R2 =0.04; OPRM1, R2 =0.04; CHRM, R2 =0.0001) (PNG 1061 KB) [file 12035_2024_4334_MOESM2_ESM.png]
